# Metabolic and transcriptional effects of bazedoxifene/conjugated estrogens in a model of obesity-associated breast cancer risk

**DOI:** 10.1172/jci.insight.182694

**Published:** 2025-03-06

**Authors:** Erin D. Giles, Katherine L. Cook, Ramsey M. Jenschke, Karen A. Corleto, Danilo Landrock, Tara N. Mahmood, Katherine E. Sanchez, Alina Levin, Stephen D. Hursting, Bruce F. Kimler, Barry S. Komm, Carol J. Fabian

**Affiliations:** 1 School of Kinesiology, and; 2 Rogel Cancer Center, University of Michigan, Ann Arbor, Michigan, USA.; 3 Departments of Surgery and Cancer Biology, Wake Forest University School of Medicine, Winston-Salem, North Carolina, USA.; 4 Interdisciplinary Program in Genetics, and; 5 Department of Nutrition, Texas A&M University, College Station, Texas, USA.; 6 Department of Nutrition and Nutrition Research Institute, and; 7 Lineberger Comprehensive Cancer Center, University of North Carolina, Chapel Hill, North Carolina, USA.; 8 Department of Radiation Oncology, University of Kansas Medical Center, Kansas City, Kansas, USA.; 9 Komm Pharma Consulting, LLC, Philadelphia, Pennsylvania, USA.; 10 Department of Internal Medicine, Divisions of Medical Oncology and Precision Prevention, University of Kansas Medical Center, Kansas City, Kansas, USA.

**Keywords:** Metabolism, Oncology, Adipose tissue, Breast cancer, Obesity

## Abstract

Many risk-eligible women refuse tamoxifen for primary prevention of breast cancer due to concerns about common side effects such as vasomotor symptoms. Tamoxifen may also induce or worsen insulin resistance and hypertriglyceridemia, especially in women with obesity. The combination of bazedoxifene and conjugated estrogens (BZA/CE) reduces vasomotor symptoms and is currently undergoing evaluation for breast cancer risk reduction. However, the impact of BZA/CE on insulin resistance and metabolic health, particularly in those with excess adiposity, is understudied. Here, we examined the effects of obesity on response to BZA/CE in a rat model of breast cancer risk using older ovary-intact rats. Female Wistar rats received carcinogen to increase mammary cancer risk and were fed a high-fat diet to promote obesity. Lean and obese rats were selected based on adiposity, and then randomized to BZA/CE or vehicle for 8 weeks. BZA/CE reduced adiposity, enriched small (insulin-sensitive) mammary adipocytes, increased the abundance of beneficial metabolic gut microbes (*Faecalbaculum rodentium* and *Odoribacter laneus*), and reversed obesity-associated changes in lipids and adipokines. BZA/CE also reversed obesity-induced mammary enrichment of cell proliferation pathways, consistent with risk-reducing effects. Together, these data support the use of BZA/CE to improve metabolic health and reduce breast cancer risk in individuals with obesity.

## Abstract 2

In a rat model of perimenopause and obesity, the combination of bazedoxifene+conjugated estrogens had beneficial effects on metabolic health while also reducing markers of breast cancer risk.

## Introduction

Approximately 25% of US women between the ages of 45 and 60 are at sufficient risk for breast cancer to be considered for risk reduction via endocrine therapy, such as tamoxifen. However, the majority of risk-eligible women resist the use of tamoxifen for primary prevention due to concerns about side effects, particularly common ones such as vasomotor symptoms (1–4). Many women in menopause transition, which typically begins in the mid-40s (5, 6), are already experiencing vasomotor symptoms and are more likely to be looking for relief rather than risking worsening symptoms with tamoxifen (7).

Menopause transition is also associated with an increase in visceral adiposity and insulin resistance, which in turn increases the risk for breast cancer (8–12). Approximately 30% of adult women in the United States are known to have insulin resistance, with a dramatic increase at menopause due to a fall in levels of estrogens (11, 12). At both standard, as well as reduced doses, tamoxifen may induce or worsen hypertriglyceridemia, hepatic steatosis, and insulin resistance, especially in women who are overweight or obese (13, 14). In contrast, hormone replacement therapy with an estrogen with or without a progestin in peri- and postmenopausal women is likely to reduce insulin resistance as well as relieve menopausal symptoms (15–17). Estrogens also have beneficial anticancer effects. For example, the Women’s Health Initiative study demonstrated that conjugated estrogens (CE) alone in women with a prior hysterectomy was associated with a 22% relative reduction in breast cancer; this is in contrast with an increase in breast cancer risk when the progestin medroxyprogesterone acetate was added to CE in women with a uterus (7, 18).

Bazedoxifene (BZA) is a selective estrogen receptor (ER) modulator with selective ER downregulator–like properties. BZA is antiestrogenic in both the breast and uterus; thus, when administered with an estrogen in women with an intact uterus, a progestin is unnecessary (15–17). BZA (20 mg) combined with 0.45 mg CE (BZA/CE) as Duavee is FDA-approved for relief of hot flashes and prevention of osteoporosis (19, 20). In a single-arm pilot study, this drug combination has also shown favorable modulation of mammographic density and several tissue biomarkers associated with risk for breast cancer (21). BZA/CE is currently being evaluated in a phase IIB primary breast cancer prevention trial (22).

Studies in ovariectomized mice suggest a favorable effect of BZA/CE on insulin resistance (23). Improvement in measures of insulin resistance was not observed in a study of 12 women given the drug for 8 weeks; however, given the small sample size and short duration, additional studies are needed (24). Thus, our goal in this preclinical study was to assess the effects of BZA/CE on insulin resistance parameters, adipose distribution, and biomarkers of metabolic health in both lean and obese older rats to approximate the perimenopausal human condition. In addition, we assessed the impact of BZA/CE on mammary tissue gene expression. Together, these data will help inform biomarker assessment in our ongoing phase IIB clinical trial of BZA/CE for primary prevention of breast cancer.

## Results

### Body weight and body composition.

**Figure 1:**
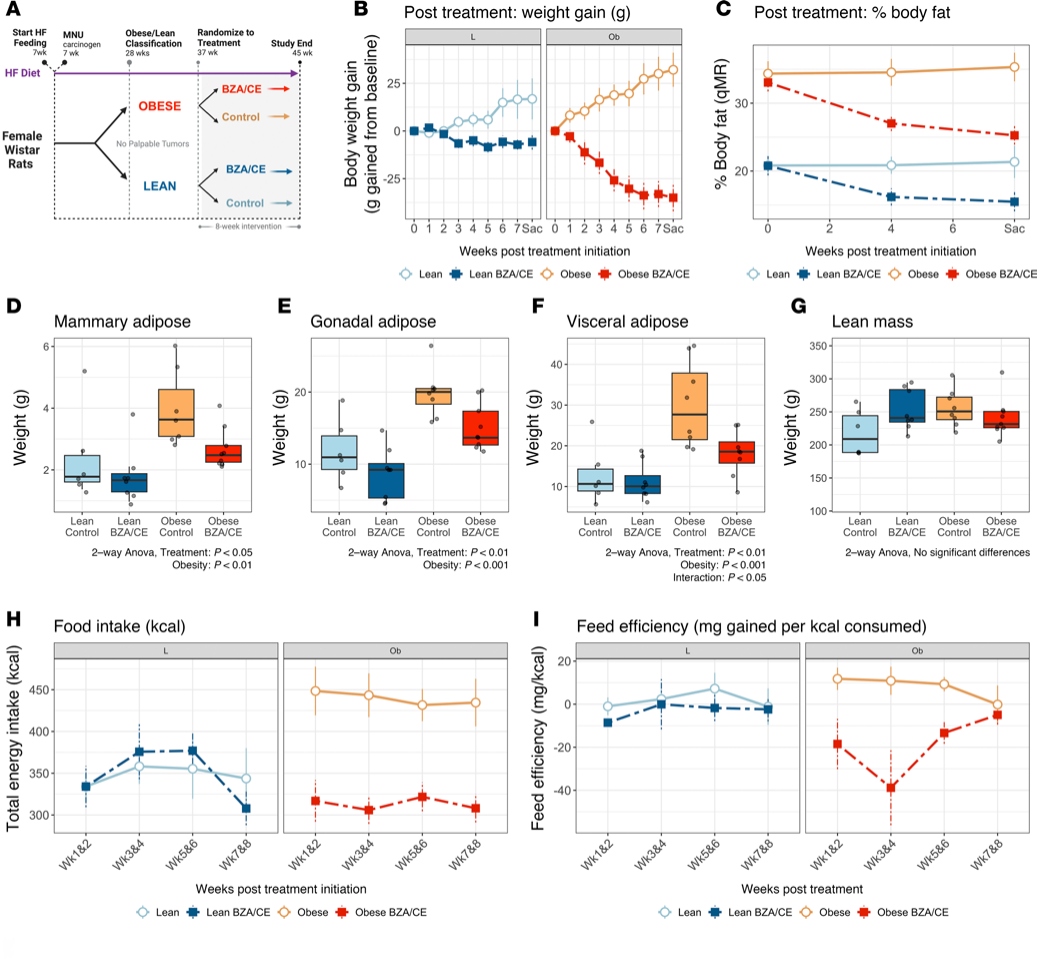
BZA/CE decreases body weight, adiposity, and food intake in lean and obese rats. (**A**) Study design diagram depicting times at which rats were given high-fat diet (HF), carcinogen (*N*-methyl-*N*-nitrosourea, MNU), and randomized to BZA/CE treatment or vehicle control. (**B**) Cumulative weight gain across the 8-week treatment period. (**C**) Percentage body fat across the treatment period, measured by qMR. (**D**–**F**) Mass of mammary, gonadal, and visceral adipose depots at the end of study. (**G**) Total lean mass, as measured by qMR, at the end of study. (**H** and **I**) Energy intake (kcal) and feed efficiency (calculated as mg of weight gained per kcal consumed) across the 8-week treatment period. Data in line graphs represent mean ± SEM; data in box-and-whisker plots represent mean ± interquartile range. Data were analyzed by 2-way ANOVA, with main effects of adiposity, treatment, and their interaction. For all graphs, *n* = 6 lean control, 8 lean BZA/CE, 8 obese control, and 9 obese BZA/CE rats.

The general study design is shown in Figure 1A. All rats were fed a high-fat diet (HFD) to induce obesity, given a single i.p. injection of carcinogen to increase DNA damage in the mammary gland during the period of mammary development, and randomized to oral BZA/CE (Duavee) or vehicle for 8 weeks.

We first examined the impact of BZA/CE on body weight and fat distribution in lean and obese rats. Treatment with BZA/CE significantly reduced both body weight (Figure 1B) and percentage body fat (Figure 1C) in all animals over the 8-week treatment period. These effects were more profound in obese rats compared with lean. At the end of the study, obese rats treated with BZA/CE weighed 19% less than obese control animals (351 vs. 436 g; *P* < 0.05), while in lean rats, BZA/CE only lowered body weight by 6% (305 vs. 325 g; *P* < 0.05). Similarly, total percentage body fat was reduced in all animals receiving drug, but the BZA/CE-induced reduction in body fat was greater in obese rats compared with lean (Figure 1C).

Regional adipose distribution was also assessed at the end of the study. Obese control rats had significantly greater adipose mass in all depots measured (mammary, gonadal, and visceral adipose) when compared with their lean counterparts (*P* < 0.05). BZA/CE treatment significantly reduced adipose accumulation in these depots (Figure 1, D–F; 2-way ANOVA, effect of treatment, *P* < 0.05), with a greater impact on reducing visceral adipose depot mass in obese rats compared with lean. Importantly, we saw no significant difference in lean mass between groups (Figure 1G), suggesting that the BZA/CE-induced weight loss was due to a reduction in fat mass.

### Food intake and feed efficiency.

BZA/CE had no significant impact on food intake in lean rats, but in obese rats, BZA/CE significantly reduced caloric intake versus obese controls (mean caloric intake of 315 vs. 435 kcal/wk; *P* < 0.001; Figure 1H). We also found a significant reduction in the storage efficiency of ingested fuels (lower feed efficiency) in obese BZA/CE rats during the first 4 weeks of BZA/CE treatment, but feed efficiency returned to that of control rats by the end of the 8-week treatment period (Figure 1I).

### Measures of insulin resistance.

**Figure 2:**
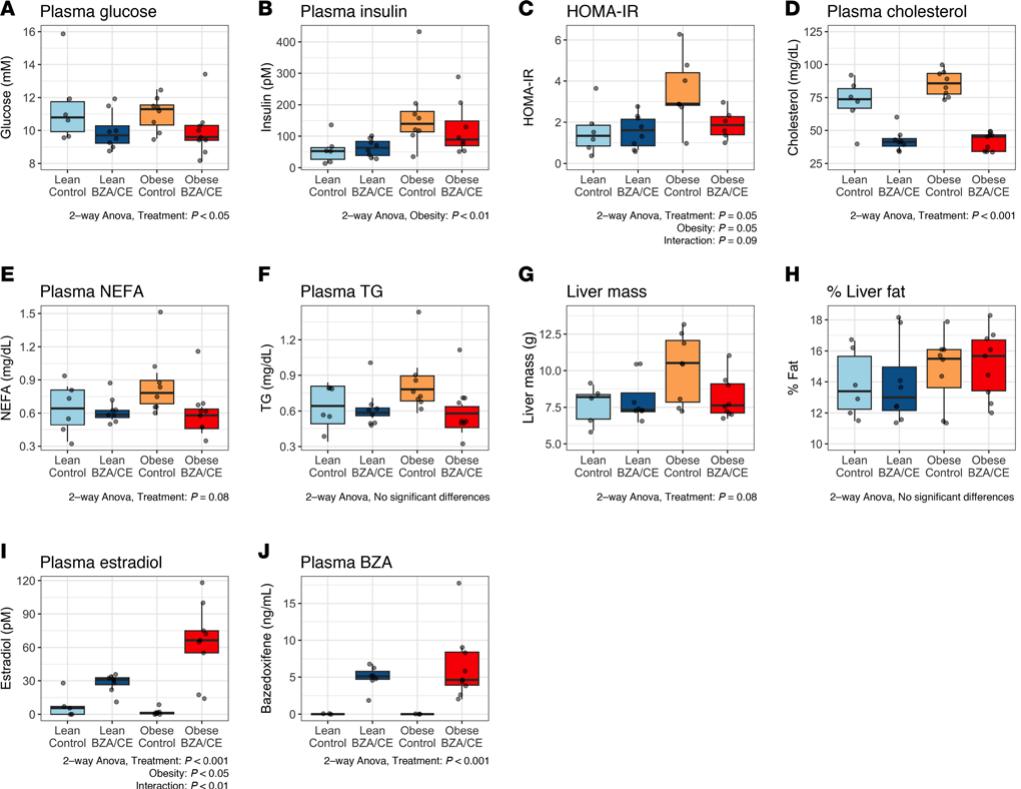
BZA/CE improves markers of insulin resistance and metabolic health. (**A**) Glucose and (**B**) insulin were measured in end-of-study plasma samples and used to calculate (**C**) HOMA-IR. Plasma levels of (**D**) cholesterol, (**E**) non-esterified free fatty acids (NEFAs), and (**F**) triglycerides (TG) were measured at end-of-study plasma using colorimetric assays. (**G**) Liver mass and (**H**) percentage fat in liver (by qMR) at end of study. Plasma levels of (**I**) estradiol and (**J**) BZA measured in end-of-study plasma using ELISA and mass spectrometry, respectively. All data are presented as mean ± interquartile range and were analyzed by 2-way ANOVA, with main effects of adiposity, treatment, and their interaction. In **A**–**H**, *n* = 5–6 lean control, 8 lean BZA/CE, 8 obese control, and 8–9 obese BZA/CE rats. In **J**, *n =* 3 lean control, 7 lean BZA/CE, 3 obese control, and 9 obese BZA/CE.

Glucose, insulin, homeostatic model assessment for insulin resistance (HOMA-IR), and hepatic lipid accumulation were assessed at the end of the 8-week intervention as markers of whole-body insulin resistance. We saw no difference in fasting glucose levels based on adiposity status (lean vs. obese), but rats treated with BZA/CE had significantly lower glucose levels than controls (Figure 2A). Plasma insulin was significantly higher in obese rats compared with lean, and while we saw a trend for lower insulin in obese rats treated with BZA/CE, this did not reach significance (Figure 2B). HOMA-IR was significantly higher in obese rats compared with lean (*P* < 0.05; 2-way ANOVA lean vs. obese), with a trend for reduction in HOMA-IR in obese rats treated with BZA/CE compared with obese controls (Figure 2C; 4.68 vs. 3.13, *P* = 0.09). BZA/CE had additional benefits on circulating metabolic factors, including a significant reduction in plasma cholesterol, and a trend for lower non-esterified free fatty acids (NEFAs) (*P* = 0.08) (Figure 2, D–F). BZA/CE had minimal effect on the liver; there was a trend for lower liver weight in BZA/CE-treated rats compared with untreated controls (*P* = 0.08, Figure 2G), and importantly, BZA/CE had no impact on liver fat accumulation in either lean or obese rats (Figure 2H).

### Circulating estradiol and BZA.

Plasma estradiol and BZA were measured in end-of-study plasma samples. As expected, estradiol was significantly higher in BZA/CE-treated rats compared with controls, and levels in obese BZA/CE-treated rats were higher than in their lean BZA/CE-treated counterparts (Figure 2I). Circulating levels of BZA were increased with BZA/CE treatment, with no difference between lean and obese rats (Figure 2J).

### Adipokines and adipocyte size.

**Figure 3:**
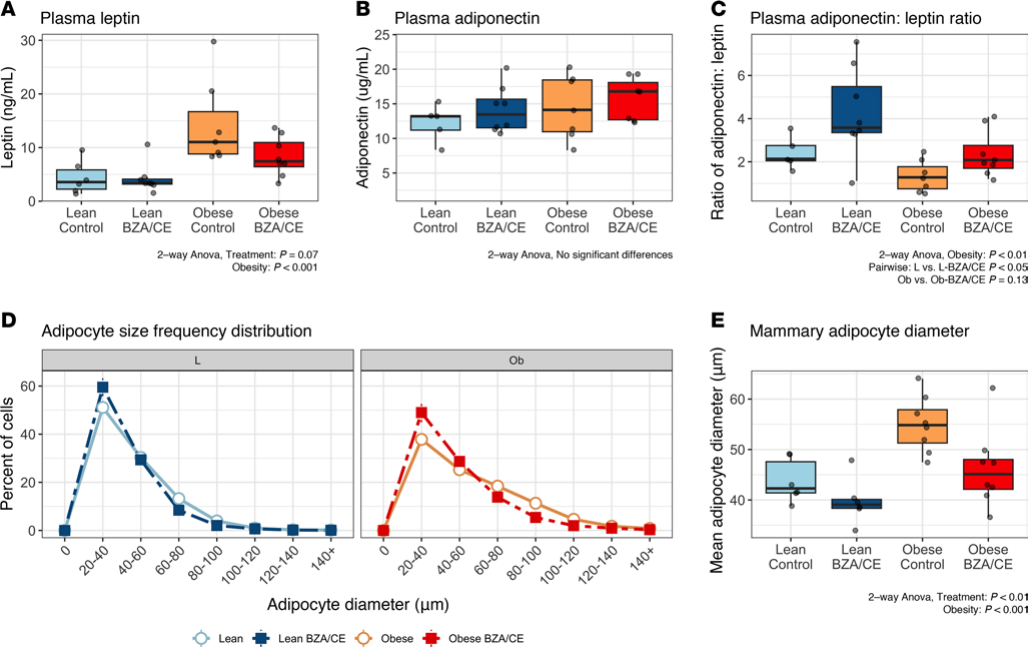
BZA/CE increases adiponectin/leptin ratio and reduces the size of mammary adipocytes. (**A**) Plasma leptin, (**B**) adiponectin, and (**C**) the ratio of adiponectin to leptin in end-of-study plasma (*n* = 6 lean control, 8 lean BZA/CE, 7–8 obese control, and 8 obese BZA/CE rats). (**D**) Cell size distribution of subcutaneous/mammary adipocytes in lean and obese BZA/CE or control rats at the end of study (*n* ≥ 1200 cells per tissue, with *n* = 6 lean control, 6 lean BZA/CE, 8 obese control, and 8 obese BZA/CE rats per group). Differences in adipocyte cell frequency distribution were examined by χ^2^ analysis. (**E**) Mean adipocyte diameter of cells in subcutaneous/mammary depots at the end of study (*n* = 6 lean control, 6 lean BZA/CE, 8 obese control, and 8 obese BZA/CE rats per group). Box-and-whisker plots indicate mean ± interquartile range and were analyzed by 2-way ANOVA, with main effects of adiposity, treatment, and their interaction. For **C**, Bonferroni-adjusted pairwise comparisons are also shown.

Examining plasma adipokines and adipose health, we found that leptin was significantly increased with obesity, and tended to be lowered with BZA/CE (Figure 3A), again primarily driven by a reduction in leptin in the obese BZA/CE-treated rats, reflecting the BZA/CE-associated reduction in adipose mass as described above. Adiponectin levels were not significantly altered by adiposity or treatment (Figure 3B). However, the adiponectin-to-leptin ratio, a functional biomarker of insulin sensitivity (25–27), was higher in lean rats versus obese, and suggested improvement with BZA/CE. This effect of BZA/CE reached statistical significance in the lean rats treated with BZA/CE versus control, but the increase in the BZA/CE-treated obese rats versus obese control (Figure 3C) was not statistically significant. Finally, we analyzed mammary adipocyte size, as small adipocytes are more insulin sensitive than larger cells (28). The proportion of small adipocytes was increased in lean rats compared with obese (Figure 3D). Furthermore, BZA/CE treatment increased the proportion of small adipocytes (20–40 μm diameter) and reduced the proportion of larger (60–120 μm diameter) cells, especially in obese rats. This resulted in a significantly higher mean adipocyte diameter in lean rats compared with obese, and a significant reduction in mean adipocyte size with BZA/CE treatment (Figure 3E).

### Microbiome.

**Figure 4:**
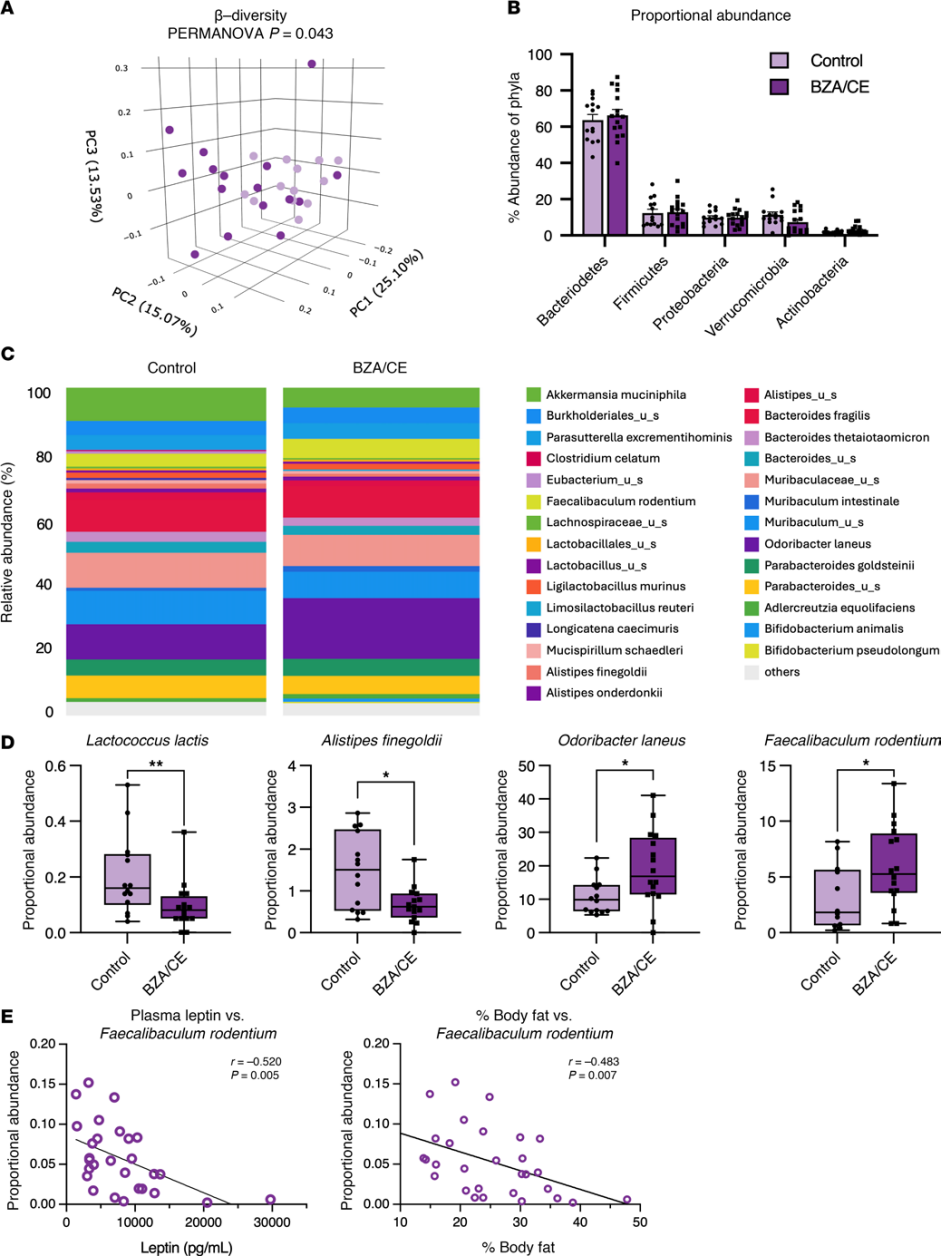
Impact of BZA/CE on the gut microbiome. (**A**) Principal coordinate analysis (PCoA) of β-diversity (Bray-Curtis dissimilarity index) showing no difference between BZA/CE-treated versus control rats. (**B**) Proportional abundance of each microbiome phylum in control and BZA/CE-treated rats. Bar graph indicates mean ± SEM. (**C**) Relative proportional abundance of bacterial species in control and BZA/CE-treated rats. Each colored box in the bar graph represents a bacterial taxon and the height of the box represents the relative abundance of that bacteria within the sample. “Other” represents lower abundance taxa. (**D**) Proportional abundance of key bacterial species known to be linked to obesity/metabolic health that were also altered with BZA/CE treatment. Box-and-whisker plots indicate mean ± interquartile range, and were analyzed by Mann-Whitney test. *P < 0.05, **P < 0.01. (**E**) Correlation between the proportional abundance of *Faecalbaculum rodentium* and end-of-study plasma leptin and percentage body fat; Spearman’s correlation coefficients are shown. All analyses represent *n* = 13–14 control and 16 BZA/CE-treated rats.

BZA/CE treatment did not result in significant differences in measures of microbiome α-diversity (Chao1 or Shannon α-diversity; data not shown). In contrast, rats administered BZA/CE had a significant shift in β-diversity (Bray-Curtis dissimilarity index), regardless of adiposity. The principal coordinate analysis (PCoA) highlighting this effect is shown in Figure 4A (PERMANOVA, *P* = 0.043). Given that we saw no significant differences based on adiposity, the lean and obese groups were combined for subsequent microbiome analyses, and again we found no significant differences in proportional abundance at the phylum level between control and BZA/CE-treated animals (Figure 4B). We then assessed the relative proportional abundance of bacterial species in control and BZA/CE-administered rats (Figure 4C). Regardless of adiposity (lean/obese), rats treated with BZA/CE had a decreased proportional abundance of *Lactococcus lactis* and *Alistipes finegoldii* and an increased proportional abundance of *Odoribacter laneus* and *Faecalbaculum rodentium* (Figure 4D). Proportional abundance of *Faecalbaculum rodentium* negatively correlated with both circulating leptin levels at the end of the study and percentage body fat at the same time point (Figure 4E), implicating these BZA/CE-regulated gut microbes in potential favorable metabolic outcomes.

### Mammary gland gene expression.

**Figure 5:**
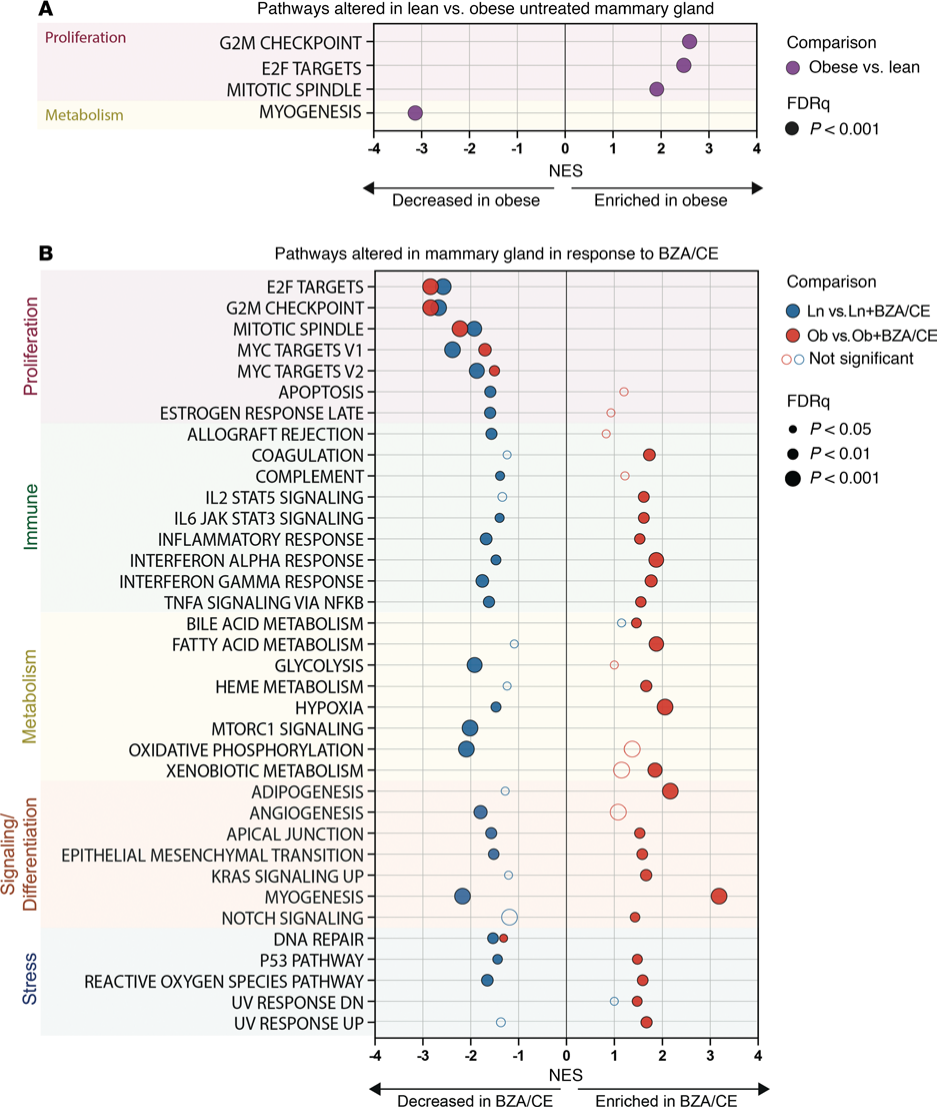
Impact of BZA/CE on mammary gland gene expression. Mammary glands from lean and obese, untreated, and BZA/CE-treated rats were subjected to gene expression microarray analysis. (**A**) GSEA MSigDB Hallmark gene sets significantly enriched/decreased in obese untreated rats compared with lean untreated. (**B**) GSEA MSigDB Hallmark gene sets significantly enriched/decreased in BZA/CE-treated rats compared with untreated controls, with lean shown in blue, obese shown in red. The size of each symbol indicates the level of significance. NES, normalized enrichment score; significance was defined as an NES > 1.5 with FDR *q* < 0.05. Open circles are provided for comparison purposes, indicating the direction and fold change of genes that did not meet our predefined levels of significance. *n* = 5 samples per group (lean control, lean + BZA/CE, obese control, and obese + BZA/CE).

Finally, we examined the effects of adiposity and BZA/CE on gene expression in the mammary gland. In a comparison of samples from lean versus obese untreated animals, we found 114 differentially expressed genes, with obese control rats showing enrichment of proliferation-associated pathways and reduction in those related to myogenesis, when compared with lean controls (Figure 5A).

**Figure 6:**
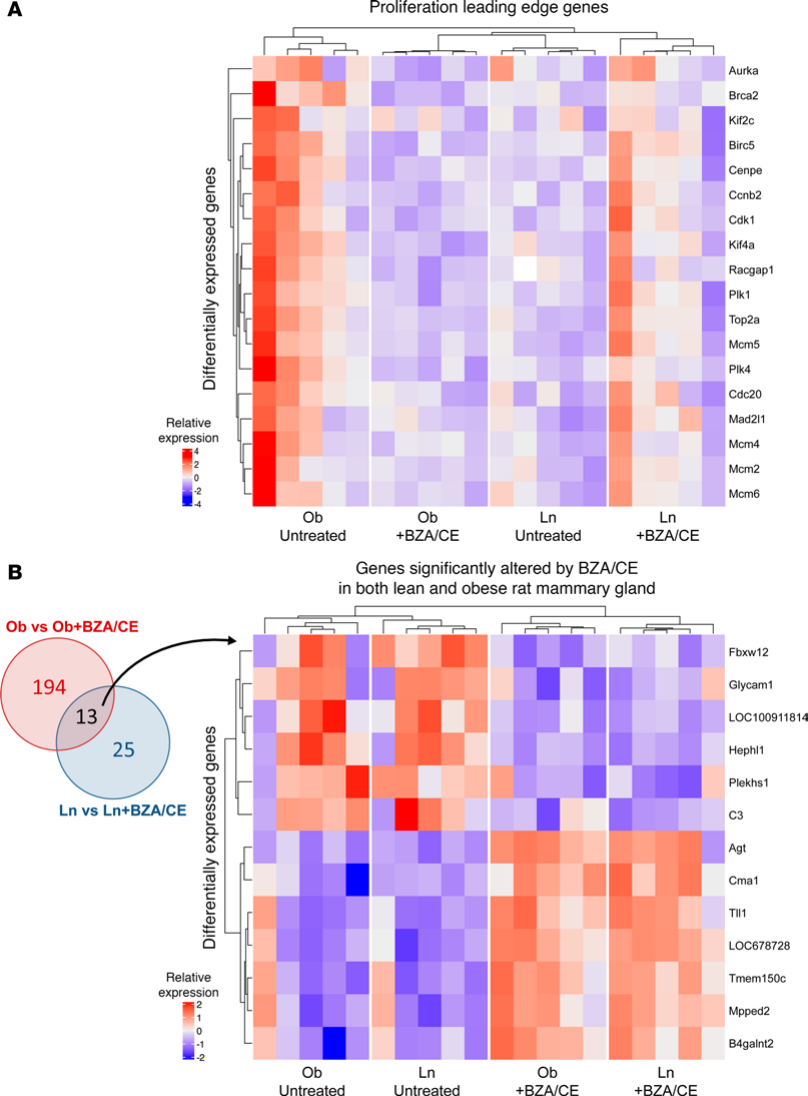
Differentially expressed genes in mammary glands of lean and obese rats with and without BZA/CE treatment. (**A**) Heatmap of expression of leading-edge genes from proliferation-related pathways from Figure 5B. (**B**) Left: Venn diagram showing the number of differentially expressed genes in the mammary gland of obese versus obese BZA/CE compared with lean versus lean BZA/CE. Right: Heatmap showing expression of 13 genes that were differentially expressed in both obese versus obese BZA/CE and lean versus lean BZA/CE. *n* = 5 samples per group (lean control, lean BZA/CE, obese control, and obese BZA/CE).

When comparing BZA/CE-treated rats versus untreated controls, BZA/CE treatment was associated with a significant reduction in proliferation-associated pathways in both lean and obese treated rats (Figure 5B); other pathways altered with BZA/CE treatment differed by obesity status (Figure 5B). Immune, metabolism, signaling, and stress pathways were enriched with BZA/CE treatment in the obese, whereas these pathways were reduced with BZA/CE treatment in the lean (Figure 5B). Leading-edge analysis revealed core genes that account for the decrease in proliferation-related pathways; a heatmap showing differential expression of these genes across all groups is shown in Figure 6A. Finally, we identified 13 genes that were differentially expressed in response to BZA/CE in mammary glands from both obese and lean rats (Figure 6B). These changes included BZA/CE-mediated downregulation of *Plekhs1*, a biomarker of glucose intolerance (29) known to be associated with other cancers (30–33); *C3*, an estrogen-dependent gene involved in the activation of the complement system (34); and *Glycam1*, a prolactin- and progestin-dependent gene (35). Genes upregulated by BZA/CE included *Agt* and *Cma1*, members of the angiotensin pathway, and *Tll1* which encodes a zinc-dependent metalloprotease.

## Discussion

In this translational study, we assessed the effects of BZA/CE on the mammary gland and several measures of metabolism in lean and obese older ovary-intact rats. Older ovary-intact rats were specifically used to simulate the early menopause transition in women, and exposure to carcinogen was used to simulate an increased mammary cancer risk, without the development of overt mammary tumors. Much of the research on BZA/CE to date has been in the postmenopausal setting, and the few preclinical studies completed in ovary-intact mice have focused on endometriosis treatment (36). Clinical data in premenopausal women are similarly sparse, but a small case series reported that this drug combination is well tolerated in premenopausal women suffering from endometriosis (37), suggesting that it should be safe in women who are still technically premenopausal but suffering from vasomotor symptoms.

Here, our data demonstrate that BZA/CE reduced expression of proliferation-related genes in the mammary glands of both lean and obese rats. This was similar to findings from non-obese ovariectomized non-human primates, where BZA/CE reduced ER expression, cell proliferation, epithelial density, and lobular enlargement in normal breast tissue compared with CE alone (38). Furthermore, our analysis revealed several genes that could be evaluated as potential biomarkers of BZA/CE effectiveness in ongoing clinical studies. In sum, our finding that BZA/CE retains antiproliferative effects in ovary-intact rats both with and without obesity further extends the potential applicability of this drug combination for cancer prevention and/or treatment.

BZA/CE also demonstrated favorable metabolic effects in obese rats, with a reduction in body weight; reduced mammary, gonadal, and visceral adipose tissue mass; reduced mammary adipocyte size; an increase in the ratio of adiponectin to leptin; reduced glucose and cholesterol; and trends towards lower insulin, HOMA-IR and free fatty acids. These trends would likely have been significant with a larger sample size.

As the gut microbiome is a key variable modulating metabolic outcomes, we investigated the impact of drug administration on this parameter. In congruence with a previous study in mice (39), we did not observe significant changes in α-diversity measurements. However, we did observe that drug administration resulted in significant differences in β-diversity. Specifically, rats treated with BZA/CE had reduced *Lactococcus lactis* and *Alistipes finegoldii and* increased *Odoribacter laneus* and *Faecalbaculum rodentium* proportional abundances. A previous clinical study investigating associations between the gut microbiome and breast cancer found that women with breast cancer had higher proportional abundance of *Alistipes* spp. when compared with non-cancerous controls (40), suggesting that the BZA/CE-mediated reduction of this population may be beneficial for anticancer outcomes.

Our finding that *Faecalbaculum rodentium* was inversely correlated with both plasma leptin and adiposity suggests that it may play a role in reducing obesity. This is supported by previous studies showing fecal microbiome transfers from calorie-restricted mice, enriched in *Faecalbaculum* spp., reduced HFD-induced weight gain (41). Moreover, additional studies have shown that *F*. *rodentium* and its human analogous species (*Holdemanella biformis*) display direct anticancer outcomes via short-chain fatty acid production (42), further supporting the beneficial effects of these microbes.

Finally, *Odoribacter laneus* has been previously shown to improve metabolic outcomes in obese mice, supporting the potential benefit of the increase in this bacterium in our study (43). These results are also similar to the beneficial metabolic effects reported with BZA/CE in ovariectomized mice (23, 44) and together suggest that favorable modulation of the microbiome could contribute to beneficial metabolic effects and reduction in adiposity observed in this study.

### Comparison with tamoxifen.

Although both tamoxifen and BZA (even when concomitantly administered with CE) reduce benign breast tissue proliferation, they differentially affect metabolism. Tamoxifen can increase triglyceride levels, especially in overweight individuals, and promote fatty liver infiltration and nonalcoholic steatohepatitis (45–47). In contrast, our current data demonstrate that combining CE with BZA has the opposite effect, lowering triglycerides, cholesterol, and NEFAs. BZA alone reduces LDL without an effect on triglycerides (48). However, since estrogens alone can increase triglycerides (49), the combination of BZA/CE is balancing these two opposing effects. In human studies, combining BZA and CE has been shown to increase HDL and reduce LDL, thereby improving cholesterol similarly to this study, but results in a small increase in triglycerides (50). BZA/CE did not induce hepatic lipid accumulation in this study, and a previous preclinical study found that BZA/CE reduced steatosis in ovariectomized animals where basal liver dysfunction was high (44). Thus, together with previous findings, our data suggest that BZA/CE is likely superior to tamoxifen for hepatic outcomes, particularly in the context of underlying obesity.

### Relevant ongoing clinical trials with BZA/CE.

There are currently 2 ongoing clinical trials examining BZA/CE clinically. The PROMISE study is a window of opportunity trial comparing the change in breast tissue proliferation and estrogen-responsive genes with 4 weeks of BZA/CE compared to placebo in women with ER-positive ductal carcinoma in situ (ClinicalTrials.gov NCT02694809). Our group (PI, CJF) is conducting a phase IIB trial of 6 months of BZA/CE versus waitlist control in peri- and early postmenopausal women with both vasomotor symptoms and increased risk for breast cancer (ClinicalTrials.gov NCT04821141). The primary endpoint is the change in mammographic fibroglandular volume between the 2 arms but will also measure changes in benign breast proliferation and estrogen response gene and protein expression. Based on the favorable metabolic findings in this preclinical study, we are also measuring the change in total and visceral adipose tissue mass and insulin sensitivity as exploratory endpoints.

### Study limitations.

One of the main limitations of this study, largely due to budget constraints, was our assessment of the combined impact of BZA and CE without additional study arms assessing each agent alone. In the United States, BZA is not approved for single-agent use, and thus we primarily focused on the future translation of these data to humans where BZA and CE are used in combination. While this limits our ability to discern whether specific outcomes of our study are due to BZA, CE, or combined effects of both agents, a previous study by Kim and colleagues (44) performed a detailed analysis of the metabolic effects of BZA, CE, and BZA/CE in ovariectomized mice during HFD feeding. When assessing markers of glucose homeostasis and insulin tolerance, they found that BZA as a single agent had similar effects as E2 and CE as single agents, and when given in combination, there were no apparent additive effects. While Kim et al. assessed the metabolic effects of each agent alone, they did so in ovariectomized mice (as compared with rats in the current study), they did not compare lean versus obese phenotypes, nor did they examine the microbiome or mammary gene expression as we did in the current study, making the two studies distinct.

A limitation of our genomic analysis of the mammary adipose is that it was performed on whole adipose tissue, which contains many cell types (adipocytes, epithelial cells, immune cells, fibroblasts, etc.); thus, we cannot identify the individual cell types responsible for the gene expression changes observed in this study. Future studies using single-cell RNA-sequencing analysis of the mammary gland in response to BZA/CE would be needed to fully understand the individual impact of this drug combination on individual cell types.

### Conclusion.

In summary, our preclinical data support BZA/CE as an agent with potential beneficial effects on breast cancer risk reduction and improvements in metabolic health in women with obesity at high risk for breast cancer who are undergoing the menopause transition. It will be prudent to explore the expression of proliferation-related markers and metabolic outcomes in ongoing clinical trials of BZA/CE, and examine the additional impact of adiposity on change in systemic and breast molecular risk biomarkers in these studies.

## Methods

### Sex as a biological variable.

Given our interest in understanding alternatives to tamoxifen in women with obesity entering the menopause transition, this preclinical study used a model of diet-induced obesity and ER-positive breast cancer risk/progression in female rats.

### Preclinical model.

This study used our well-characterized rat model of diet-induced obesity and ER-positive breast cancer risk/progression, as previously described (51, 52). Briefly, 6-week-old female Wistar rats were obtained from Charles River Laboratories. Upon arrival, they were individually housed in wire-bottom cages to limit physical activity. Rats were also given ad libitum access to a purified HFD (46% kcal from fat; Research Diets, D12344) to induce obesity in this genetically susceptible strain (53–56). Rats were on a 12-hour light/dark cycle with ad libitum access to the HFD and water for the duration of the study.

The study design is shown in Figure 1A. At 7 weeks of age, 100 rats received a single i.p. injection of the carcinogen *N*-methyl-*N*-nitrosourea (50 mg/kg; MRIGlobal) to increase DNA damage in the mammary gland during the period of mammary development. In the current study, we were interested in studying metabolic and mammary gland–specific effects in older ovary-intact animals in the absence of overt cancer burden. Thus, rats in which tumors were detected early in the study were used in a separate study (*n* = 59; forthcoming). The remaining non–tumor-bearing rats (*n* = 41) were used for the current study.

At 28 weeks of age, the non–tumor-bearing rats (*n* = 41) were ranked by their percentage body fat; those in the top and bottom approximately 40% of adiposity were classified as obese and lean, respectively. Rats from the middle group (*n* = 6) were removed from the study, as were 4 additional animals (2 of whom were sick and 2 with off-target tumors). At 37 weeks of age, the remaining lean (*n* = 14) and obese (*n* = 17) rats were then randomized to receive either a daily oral dose of Duavee (3 mg BZA + 0.07 mg CE/kg body weight) for 8 weeks, or vehicle control. BZA/CE was prepared by grinding Duavee tablets using a mortar and pestle. The resulting powder was dissolved in a small volume of corn oil, and then mixed with Nutella spread for delivery to the animals (3 Duavee pills + 50 μL corn oil + 10 g Nutella). Animals were provided the experimental agent immediately prior to the start of the dark cycle, and the drug/vehicle mixture was consumed in its entirety. This achieved an average plasma BZA concentration of 5.83 ± 0.94 ng/mL at the end of the study, with no difference between lean and obese rats.

Rats were euthanized by exsanguination under anesthesia following 8 weeks of treatment. At the study end, fat pads were weighed to determine regional fat distribution, and blood and tissues were collected and processed as described below.

### Body weight, body composition, and food intake.

Body weight and food intake were monitored weekly throughout the study, as previously described (53, 57). Feed efficiency was calculated as the change in body weight (mg) per kcal of food consumed over the same period. Body composition was determined by quantitative magnetic resonance (qMR; EchoMRI; Echo Medical Systems) at 14, 18, 22, and 28 weeks of age, at the initiation of BZA/CE treatment, every 2 weeks after treatment, and again at the time of euthanasia.

### Plasma measurements.

Tail vein blood was collected immediately prior to initiation of BZA/CE treatment and again at the time of euthanasia. Blood was drawn during the latter part of the light cycle following a 6-hour fast; plasma was isolated and stored at −80°C. Plasma insulin, adiponectin, and estradiol were measured by ELISA (Alpco; 80-INSRT-E01, 22-ADPRT-E01, and 55-ESTRT-E01, respectively). Colorimetric assays were used to measure plasma NEFAs [HR series NEFA-HR(2); Wako Chemicals], glucose, triglycerides, and total cholesterol (Thermo Fisher Scientific; TR15421, TR22321, and TR13521, respectively). HOMA-IR was calculated as a proxy for insulin resistance using the following formula: fasting glucose (mM) × fasting insulin (pM)/405. BZA levels were analyzed by ultra-high-performance liquid chromatography–tandem mass spectrometry (University of Kansas Cancer Center Clinical Pharmacology Shared Resource).

### Histological staining and adipocyte cellularity.

To assess adipocyte cell size distribution and mean adipocyte diameter, sections of formalin-fixed, paraffin-embedded adipose tissue (5 μm) were stained with H&E. For each animal, one entire H&E-stained slide was scanned using a Leica Aperio CS2 slide scanner. Using the associated ImageScope software (Leica Biosystems), 5 regions of each tissue section were randomly selected for analysis. Images were exported and cell diameter and number were measured in ImageJ (58) using the Adiposoft plug-in (59). For each slide, the diameters of more than 700 adipocytes were analyzed. Data are presented as both frequency distributions and mean adipocyte diameter.

### Microbiome analysis.

Fecal samples were collected at the end of the study and snap-frozen. DNA was isolated from fecal material using the QIAGEN DNeasy PowerLyzer Powersoil Kit and 3M read-depth shotgun metagenomic sequencing was performed by CosmosID Inc. Read depth ranged from 3M–9M per sample. DNA samples were quantified using the GloMax Plate Reader System (Promega) with QuantiFluor dsDNA System (Promega) chemistry. DNA libraries were then prepared using the Nextera XT DNA Library Preparation Kit (Illumina) and IDT Unique Dual Indexes with total DNA input of 1 ng. Genomic DNA was fragmented using a proportional amount of Illumina Nextera XT fragmentation enzyme. Unique dual indexes were added to each sample followed by 12 cycles of PCR to construct libraries. DNA libraries were purified using AMpure magnetic Beads (Beckman Coulter) and eluted in QIAGEN EB buffer. DNA libraries were quantified using a Qubit 4 fluorometer and Qubit dsDNA HS Assay Kit. Libraries were then sequenced on Illumina NovaSeq X platform, 2 × 150 bp.

For analysis, a high-performance data-mining k-mer algorithm was used that rapidly disambiguates millions of short sequence reads into the discrete genomes engendering the particular sequences. The pipeline has 2 separable comparators: the first consists of a precomputation phase for reference databases and the second is a per-sample computation. The input to the precomputation phase are databases of reference genomes, virulence markers, and antimicrobial resistance markers that are continuously curated by CosmosID scientists. The output of the precomputational phase is a phylogeny tree of microbes, together with sets of variable-length k-mer fingerprints (biomarkers) uniquely associated with distinct branches and leaves of the tree. The second per-sample computational phase searches the hundreds of millions of short sequence reads, or alternatively contigs from draft de novo assemblies, against the fingerprint sets. This query enables the sensitive yet highly precise detection and taxonomic classification of microbial next-generation sequencing (NGS) reads. The resulting statistics were analyzed to return the fine-grain taxonomic and relative abundance estimates for the microbial NGS datasets. To exclude false positive identifications the results were filtered using a filtering threshold derived based on internal statistical scores that was determined by analyzing a large number of diverse metagenomes.

### Microarray analysis.

To explore the direct effects of BZA/CE on the mammary gland, we measured differential gene expression using Affymetrix microarrays. Total RNA was isolated from flash-frozen mammary tissue using TRIzol reagent (Thermo Fisher Scientific) according to the manufacturer’s protocol. Total RNA integrity was assessed via RNA ScreenTape analysis (Agilent Technologies). The transcriptome of 5–6 RNA samples from each group (lean control, obese control, lean treated with BZA/CE, and obese treated with BZA/CE) was then profiled via the Rat Clariom S Assay HT (Thermo Fisher Scientific). Total RNA was used to synthesize fragmented and labeled sense-strand cDNA and hybridized onto the Clariom S peg plate. Samples were prepared using the GeneChip WT PLUS Reagent Kit (Affymetrix), following the manufacturer’s instructions. Fragmented and labeled cDNA was used to prepare a hybridization cocktail with the GeneTitan Hybridization Wash and Stain Kit for WT Arrays (Affymetrix). Hybridization, washing, staining, and scanning of the Clariom S peg plate was carried out using the GeneTitan MC Instrument (Affymetrix). Transcriptome Analysis Console Software version 4.0 (TAC, Thermo Fisher Scientific) was used for basic data analysis and quality control.

### Gene set enrichment analysis.

Gene set enrichment analysis (GSEA version 4.3.2; https://www.gsea-msigdb.org/gsea/index.jsp) was performed for the microarray data pairwise comparisons using the Broad Institute MSigDB Hallmark gene sets (version 2022.1) to assess changes in biological processes between mammary glands from lean versus obese rats receiving either BZA/CE or vehicle control. Normalized enrichment statistic was calculated using default settings; an FDR *q* value of less than 0.05 was considered statistically significant.

### Statistics.

Unless otherwise described, data were analyzed using R Studio (https://posit.co/download/rstudio-desktop/) or GraphPad Prism version 10. Where applicable, data are expressed as mean ± SEM. For data presented as box-and-whisker plots, the horizontal line represent the mean, box bounds represents interquartile range, (IQR), and the whiskers indicate the highest and lowest data points that are within 1.5 × IQR. All data points are shown, but any data points that fall beyond the whiskers are generally considered outliers.”. Comparisons between groups were assessed using 2-way ANOVA, examining the effect of adiposity (lean vs. obese), BZA/CE treatment (treated vs. control), and the interaction between the two. Non-normally distributed data were assessed by Kruskal-Wallis test. Relationships between variables were assessed with Spearman’s correlation coefficient. A *P* value of less than 0.05 was considered significantly different.

### Study approval.

All animal protocols were approved by the Texas A&M Institutional Animal Care and Use Committee and carried out in compliance with all regulations and guidelines.

### Data availability.

Microarray data have been deposited in the NCBI GEO under accession number GSE290908. Individual values for all other data are available online in the Supporting Data Values file.

## Author contributions

EDG, SDH, BFK, and CJF conceived the study. EDG, KLC, and SDH developed methodology. EDG, KLC, RMJ, KAC, DL, and TNM conducted experiments, EDG, KLC, and KES analyzed data. EDG, KLC, AL, SDH, BFK, and CJF wrote the original draft and edited the draft along with BSK. EDG, KLC, and KES visualized data. EDG supervised the study. EDG, KLC, SDH, BFK, and CJF acquired funding.

## Supplementary Material

Supporting data values

## Version 1 03/06/2025

In-Press Preview

## Version 2 04/22/2025

Electronic publication
